# Socioeconomic and Psychosocial Adversities Experienced by Freelancers Working in the UK Cultural Sector During the COVID-19 Pandemic: A Qualitative Study

**DOI:** 10.3389/fpsyg.2021.672694

**Published:** 2022-01-13

**Authors:** Tom May, Katey Warran, Alexandra Burton, Daisy Fancourt

**Affiliations:** Research Department of Behavioural Science and Health, Institute of Epidemiology and Health Care, University College London, London, United Kingdom

**Keywords:** COVID-19, wellbeing, work, creative careers, qualitative, mental health

## Abstract

There are concerns that the socioeconomic consequences of COVID-19, including unemployment and financial insecurity, are having adverse effects on the mental wellbeing of the population. One group particularly vulnerable to socioeconomic adversity during this period are those employed freelance within the cultural industry. Many workers in the sector were already subject to income instability, erratic work schedules and a lack of economic security before the pandemic, and it is possible that COVID-19 may exacerbate pre-existing economic precarity. Through interviews with 20 freelancers working within the performing arts, visual arts, and film and television industries, this article explores the impact of the pandemic on their working lives. Findings suggest the pandemic is affecting the psychological wellbeing of freelancers through employment loss, financial instability and work dissonance, and illustrates the need for urgent economic and psychosocial support for those employed within the cultural sector.

## Introduction

In response to COVID-19, many governments have imposed restrictions of varying stringency that have impacted on the daily lives of the general population. In the United Kingdom, this has included self-isolation, mobility constraints, and the closure of all but non-essential businesses in efforts to suppress the virus (Iacobucci, 2020). It is already evident that these containment measures have resulted in not only severe psychosocial adversities (Brooks et al., 2020; Pierce et al., 2020), but acute economic consequences for workers and businesses unable to adapt to these conditions [International Labour Organization [ILO], 2020]. Consequently, many workers have been subject to employment inactivity (e.g., furlough), income loss, or unemployment (Hensher, 2020; Witteveen and Velthorst, 2020). In the United Kingdom, 9.3 million people have entered the COVID-19 job retention scheme and another 2.7 million have claimed a self-employment income support scheme grant. Unemployment has also risen since the advent of the pandemic and is projected to increase throughout 2021 [Office for National Statistics [ONS], 2020].

Although most workers are likely to be at-risk of socioeconomic adversity during this period, one group who are particularly vulnerable are those employed freelance within the cultural sector.^1^ There are a number of reasons for this. First, the fragility of the sector before COVID-19 is widely acknowledged, including the financial precariousness of its workforce and previous susceptibility to economic shocks and recession (de Peuter, 2011; Brook et al., 2018; Banks, 2020). Major socioeconomic transformations in response to COVID-19 are therefore likely to have exacerbated any pre-existing economic vulnerability. Second, many freelancers working in the sector are reliant on cultural production sites (e.g., theatres, studios, nightclubs) for economic activity (through performances, for example). In the United Kingdom, government-mandated closures of these venues resulted in the immediate curtailing of income-facilitating activities, and there is uncertainty as to when they may reopen to allow activity to resume. As such, many freelancers have been exposed to prolonged periods of economic inactivity. Third, owing to the flexible nature of freelance employment in the cultural sector, whereby workers regularly move in and out of contracts, take on unpaid roles and work concurrently in other sectors (Throsby and Zednik, 2011), many have been ineligible for economic support in response to the pandemic. The United Kingdom COVID-19 self-employment income support scheme, for example, required a record of tax returns and for freelance work to make up at least 50% of overall income for those based in England. As a result, newly self-employed freelancers or those with “portfolio” careers were ineligible (Comunian and England, 2020).

The economic consequences of COVID-19 therefore present unique socioeconomic challenges to this workforce, including financial instability and potential unemployment. According to data from the Office for National Statistics, jobs in the arts, entertainment and recreation sector fell by 78.9% (18,000) in the year following lockdown restrictions and the ratio of vacancy numbers to positions declined from a rate of over 3.0 per 100 prior to the pandemic to 0.7 per 100 in March 2021 [Office for National Statistics [ONS], 2021]. Consequently, these industries now have the lowest ratio of vacancy numbers to employment positions in comparison to other employment sectors [Office for National Statistics [ONS], 2021]. Fifty-five thousand jobs were estimated to have been lost in music, performing and visual arts occupations between March and September 2020 (Owen et al., 2020), and, more broadly, there are now 38,000 less freelancers employed in creative professions than before the start of the pandemic (Florisson et al., 2021). Those who continue to be employed in the cultural and creative industries have also experienced a significant reduction in working hours (Owen et al., 2020). As a result, cultural workers are experiencing deleterious financial impacts: over half of performing artists surveyed (*n* = 385) during the first lockdown in the United Kingdom reported significant financial hardship following changes in working patterns and income (Spiro et al., 2021).

Unemployment and socioeconomic insecurity is a known trigger for adverse psychological consequences, including mental distress, depression, and anxiety (Marmot, 2005; Paul and Moser, 2009; Marmot et al., 2012). Theoretical explanations of unemployment’s deleterious effects accredit the loss of important functions located within paid employment to negative psychological wellbeing (Jahoda, 1981, 1982). From this view, unemployment deprives individuals of psychological needs conducive to good mental health. Jahoda (1981, 1982) suggested that work provides five “latent functions” – including time structure, social contact, collective purpose, status, and activity – beneficial to mental health and wellbeing. For example, paid work provides individuals with income and allows individuals to have a degree of control over their lives. Employment also fulfils psychological needs by providing structure and routine, social status, and a sense of identity. Additionally, employment facilitates social connections and collective purpose and opportunities to use and develop one’s skills and competencies. Relatedly, Warr (1987, 2007) identified 12 environmental characteristics fulfilled through employment (including control, skill, goals and variety, among others), the loss of which similarly impair mental health. Such accounts regard unemployment as a cause of distress on account of deprivation of such benefits. In contrast, being employed is protective of mental distress by offering several important functions that are missing if unemployed.

Empirical evidence supporting the increased incidence of stress, anxiety, depression, and poor psychological wellbeing among unemployed and economically vulnerable individuals is considerable (Murphy and Athanasou, 1999; McKee-Ryan et al., 2005; Paul and Moser, 2009). Previous literature has indicated how periods of economic recession are related to adverse mental health through factors such as job loss, financial insecurity and declining living standards (Marmot et al., 2013; Barlow et al., 2015). Moreover, perceived job loss and the prospect of financial adversity have been associated with a range of mental health symptoms during prior economic recessions (Burgard et al., 2012). There is also evidence of long-term psychological impacts stemming from unemployment, a phenomenon known as psychological scarring (Clark et al., 2001). Some have suggested that similar factors could adversely affect the health and wellbeing of unemployed workers after COVID-19 (Hensher, 2020): indeed, there is recent evidence demonstrating how COVID-19 induced economic hardship (including job loss, workload decline, and worries relating to finance) is already having adverse effects on the psychological wellbeing of the population (Witteveen and Velthorst, 2020; Wright et al., 2020).

It is widely recognised that those who are already vulnerable, whether this be due to experiencing employment precarity or for other reasons, are most at risk of health consequences during major economic changes (Marmot et al., 2013). Given their pre-existing socioeconomic vulnerabilities, it is reasonable to assume that freelancers working in the cultural sector may therefore incur significant psychosocial distress in the present context. Those working in the performing arts, visual arts, film, and television industries are particularly vulnerable, with a number of risk factors for poor mental health stemming from the precarity of artists’ careers, including feelings of anxiety, insecurity, and social isolation (Duarte, 2020; Spiro et al., 2021). More specifically, working in the performing arts industry has previously been connected to adverse mental health, with musicians experiencing stress (Parasuraman and Purohit, 2000; Gembris et al., 2018), depression, distress and anxiety (Stoeber and Eismann, 2007). Relatedly, a survey of 4877 people working in the United Kingdom film, TV, and cinema industry found that 87% experienced a mental health problem and lower levels of subjective wellbeing than the national average (Wilkes et al., 2020). This is significant given that existing psychosocial adversities may impair resilience to significant economic changes and influence the duration of unemployment (Butterworth et al., 2012). Given that freelancers working in these industries may need to withstand periods of economic inactivity in response to lockdown measures, they may therefore require targetted psychosocial support to assist with these unique support needs.

Despite the risks posed by socioeconomic adversities to the mental health and wellbeing of freelancers working in the cultural sector, little is known about the specific challenges faced by freelancers in the current context beyond quantitative enquiry [Comunian and England, 2020; Nesta Creative Industries [NCI], 2020]. Accordingly, there is a need for qualitative research that can provide in-depth data on the experiences of this population during the pandemic. This is necessary to aid our understanding of specific socioeconomic stressors among this population, and to assist in the development of future psychosocial and economic support for those at risk of financial hardship, both as the economic effects of COVID-19 persist and in future periods of economic instability. To these ends, the study aimed to explore qualitatively the impact of the COVID-19 pandemic on the working lives of freelancers working in the cultural industries in the United Kingdom, including any subsequent implications for mental health and wellbeing.

## Materials and Methods

The research employed a qualitative design using semi-structured interviews with self-employed freelancers working in the cultural sector. Participants were interviewed between July and November 2020 about their lived experiences of the pandemic, including any socioeconomic impacts resulting from COVID-19. The study formed the qualitative component of the UCL COVID-19 Social Study, a large nationwide study using survey data to explore the effects of COVID-19 and social distancing measures on adults in the United Kingdom.

### Sample and Recruitment

Convenience sampling was used to recruit self-employed freelancers working in the cultural sector from across the United Kingdom, with a particular focus on those employed within the performing arts, visual arts, museums and galleries, and film and television industries due to their greater risk of employment precarity (Duarte, 2020; Wilkes et al., 2020). In practice, this involved recruiting participants through a combination of methods including social media, personal contacts, the UKRI-funded MARCH Network, and via the UCL COVID-19 Social Study (including through its newsletter and website). The convenience approach was augmented with purposive sampling to ensure diversity of gender, age, and professional role. Eligibility was based primarily on whether the person was (or had recently been) self-employed in the cultural industries, aged over 18, and living in the United Kingdom. Participation in the research was voluntary, and all participants were provided with details either verbally or in writing about the nature and purpose of the research and what their participation would involve. Demographic details were also obtained. Ethical approval was provided by University College London Research Ethics Committee (Project ID 14895/005) and all participants provided written informed consent.

### Data Collection

Semi-structured telephone or video call interviews were conducted by TM (research fellow in social science), RC (research fellow in public health), JD (research fellow in public health and qualitative research), and SE (research assistant) using a pre-prepared topic guide that posed questions about the participant’s subjective experience(s) of the impact of the pandemic on work, mental health, and social life. The full topic guide is included in the online repository. Interviews lasted an average of 45 min, and participants were offered compensation for their time in the form of a £10 high street voucher.

With participant consent, the interviews were digitally recorded and subsequently transcribed verbatim by a professional transcription service. Data collection concluded once the theoretical saturation of themes occurred or, in other words, where instances of data emerged consistently and led to the conclusion that no further data would develop new properties, categories, or findings (Strauss, 1999).

### Data Analysis

Following anonymisation, transcripts were uploaded to NVivo version 12 software to enable computerised data analysis. A reflexive thematic approach was adopted in line with the principles of Braun and Clarke (2006, 2012), whereby one first familiarises themselves with the data before the generation and definition of codes, the searching of themes, and the production of a report. As such, five transcripts were initially read independently by two researchers (TM and KW), who coded and discussed any emerging codes that were of potential significance to the research objectives. A preliminary coding framework, informed deductively by concepts within the topic guide, was used to guide the coding of these transcripts. An inductive approach was then used to refine the framework to reflect on any emerging themes or concepts within the data and applied to the remaining transcripts by TM, who re-read transcripts and coded and synthesised text into categories, which were subsequently analysed and grouped into themes (Braun and Clarke, 2006, 2012). The qualitative research team met weekly to discuss and iteratively refine any new codes or themes emerging from this stage. This process helped to ensure that the final extracted themes were not just the personal interpretation of one team member.

## Results

Twenty participants who worked in a range of professions within the cultural sector were interviewed. Participants were aged 22–76, predominantly female (55%), and White British (55%). See Table 1 for participant characteristics.

**TABLE 1:** Characteristics of freelancers.

Number of participants	20
Profession	Contemporary dance teacher (1)
	Circus performer (1)
	Film and TV producer (2)
	Independent production associate – arts and film (1)
	Independent theatre producer and arts administrator (1)
	Musician (6)
	Playwright/theatre/opera director (3)
	Stage actor (2)
	Screen actor (1)
	Visual artist (2)
Age	22–76 (37.3)
Gender	Male (9)
	Female (11)
Ethnicity	Black or Black British (1)
	Black or Black British Caribbean (1)
	Indian (1)
	White and Black British (1)
	White and Black Caribbean (3)
	White British (11)
	White Other (2)

### Themes

**FIGURE 1:**
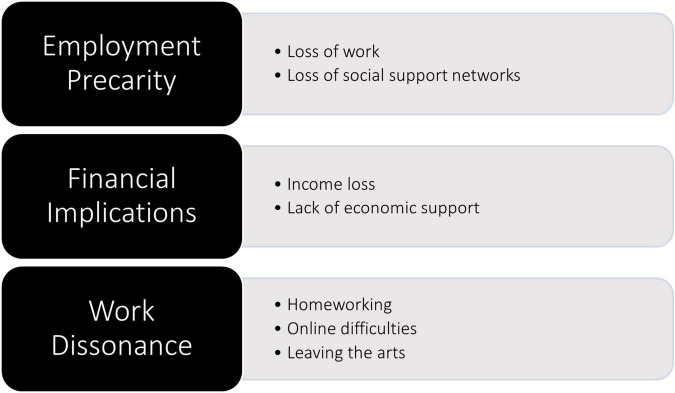
Key themes.

Interviews generated a rich body of data covering a range of issues relating to the experiences of freelancers during the pandemic, including experiences of social distancing and social isolation measures, inequalities in the sector and personal relationship changes. The data presented here, however, focus specifically on the socioeconomic changes produced by COVID-19 and their relationship with mental and social wellbeing. This issue was related to three specific themes which will be discussed in turn below: (1) employment precarity, (2) financial implications, and (3) work dissonance. These themes are presented in Figure 1, along with their respective subthemes.

### Employment Precarity

#### Loss of Work

The advent of COVID-19 resulted in government-mandated closures of theatres, music venues, nightclubs, and studios. For most participants, this resulted in the immediate loss of work (*“And then, this happened in February, and everything, all my work just disappeared overnight, pretty much,” ID1_musician*). While some participants made use of online technologies to continue work, some felt this financially unsustainable or noted limitations with remote working (see section “Online Difficulties”). Consequently, many participants expressed feelings of insecurity about the return of employment. As one musician explained:

*“I think I’m just worried about how it’s going to get back to where it was, because it’s a lot of very closed and cramped spaces within the Arts. In terms of managing to keep our distance for a long enough time, the virus isn’t going to go away…but I think it’s going to take a very long time for it to actually get back for performing to be the sole income, basically for a freelancer” (ID1_musician)*

Most participants reported that periods of fallow employment were not uncommon. It was not unusual, for example, for participants to describe periods of income instability before COVID-19 (*“I’ve had times where I’ve maybe brought in about £200 worth of income in a month,” ID2_film and tv producer*), and many spoke of the existing “precariousness,” “instability,” and “uncertainty” of freelance work. However, participants noted that the duration and severity of COVID-19 restrictions had exacerbated these worries. Combined with prolonged uncertainty surrounding the reopening of venues, pre-existing insecurities were therefore felt more acutely:

*“All the normal worries you have as a freelancer suddenly feel more real and more relevant. During this period I’ve felt very aware of the financial precariousness of being freelance, not having savings, living alone and therefore only having the one income. All those things together are fine when it all works, but one thing dropping I’ve realised, or taken more notice of, how risky that is. So I feel this sort of general sense of precariousness in the world that perhaps I didn’t feel before” (ID3_visual artist)*

The immediate loss of work was a challenging experience, particularly as some participants reported deriving enjoyment and meaning from their careers. Those who lost jobs were greatly affected and voiced insecurities and concerns. Stress and anxiety were not uncommon (*“It’s just constantly on my mind. I go through peaks and troughs of feeling quite anxious, and then telling myself that it might all get better,” ID4_theatre producer*) as were worries relating to the future of the sector (*“Sure, yes, in so many ways…the stress and anxiety about what was going to happen to my industry,” ID14_theatre producer*). Some participants also reported physical symptoms, including sleep problems that were linked to these concerns:

*“I think my sleep got messed up, especially when lockdown first kicked in. I had a lot of weird dreams going around my head. So, I think it did have an effect on that. I guess because it was just so much information to process, from news and a different way of life” (ID20_musician)*

#### Loss of Social Support Networks

The foundations of social and employability support were also affected by lockdown measures. Many participants noted the value of colleagues for emotional and social support and as a helpful resource for information sharing, often about employment opportunities. For example, one participant described how social networks were a useful conduit for obtaining and sharing news about the industry:

*“Even going to dance classes, it’s a time for us to all catch up with each other and socialise, as well as keeping up our training. Then, it’s also how we find out about what’s going on. I would say classes are a real key community event for us” (ID18_dance teacher)*

Given that participants were unable to meet colleagues face-to-face, participants noted how the loss of these networks reduced opportunities for information sharing, socialising, and networking. For example, participants were unable to meet with colleagues in work settings where much of their social life was concentrated (*“and it’s one of the features of my week that I see them every week and we have a lovely time making music together. All of that of course stopped completely and I think that the sense of being unable to go to work where my social life is centred…all that went completely,” ID6_musician*), as well as in social situations where networking often occurred (*“We would just invite people…if you wanted to meet new people and exchange ideas and have some dinner together. So that stopped completely during the pandemic,” ID19_film and tv producer*). For some, the destabilisation of these support structures impacted on their wellbeing, too:

*“I am by nature, a hugely social musician. I get my wellbeing from meeting a lot of musicians and working with them and seeing something develop from stage A to stage B and feeling perhaps some responsibility for that process. And sitting in your study, looking at your text, doesn’t do that in quite the same way” (ID6_musician)*

### Financial Implications

#### Income Loss

Without the ability to engage in cultural work, there were limited opportunities to obtain income in the CCI. Some participants therefore reported reductions in annual income in response to workload decline (*“It’s led to the almost complete elimination of my income,” ID6_musician*) or experienced substantial financial losses following the cancellation of events (*“Yes, I must have lost about £25,000 worth of work this year,” ID7_theatre director*). Due to the nature of freelance work which is often based on the scheduling of activities months in advance, these financial implications were often protracted (*“I haven’t had any income coming in for a couple of months and there won’t be anything until [3 months] from now,” ID8_musician*).

With restricted incomes, many participants reported difficulties with managing day-to-day essentials. This included concerns about the ability to pay utilities, including household bills (*“Well, financially, I went from feeling that I was really able to pay my bills, to completely cut off bare,” ID9_theatre director*) and rent (*“I can’t pay rent if I don’t work,” ID10_stage actor*). Some noted how financial disruptions had implications for material possessions (*“I would have been able to go out and buy a new car, I could’ve got myself my new phone,” ID11_independent production associate – community arts and film*) and the maintenance of existing lifestyles (*“my lifestyle’s very expensive because of all the travel…so my savings are not as secure as I’d like them to be, and that feeling of, oh my god, this could all end, is so frightening,” ID7_theatre director*). For some, the solution to these difficulties was to seek financial support from parents or partners. Often, however, this was a source of embarrassment:

*“I’m blessed my parents are helping me out with money at the moment. At the age of 47, it is embarrassing as I’ve been financially independent since the age of 18…without that, I would be getting close to struggling” (ID12_musician)*

With no immediate prospect of work and associated income returning, participants reported distress that was detrimental to their wellbeing:

*“The only pressure really has been this thing about money. It’s just money. I haven’t felt pressure in other ways. It’s been difficult, I want to be able to go into my parents’ house. I want to be able to see my son and my sister. But that’s probably been the most hardship. It’s financial and it’s that insecurity that’s the worst” (ID11_independent production associate – community arts and film)*

#### Lack of Economic Support

Participants reported a lack of economic support to assist with these financial difficulties. Many alluded to difficulties in obtaining funding and the narrow eligibility criteria required for government support. One participant, who worked in multiple freelance roles, was unable to access government support and was instead reliant on Jobseekers’ Allowance to support them through this period:

*“Then of course lockdown came and everything like that and to cut a long story short I then found out that I didn’t fit into any government scheme. I was not PAYE enough, my PAYE contract had ended at the end of January so I missed out on all the dates. I wasn’t self-employed enough because I’d had so many different PAYE contracts I hadn’t built up enough contributions to be able to qualify. So I was left with nothing apart from £74 a week contributions-based Jobseeker’s Allowance. It was crap to be quite honest” (ID11_independent production associate – community arts and film)*

Some participants implied that this lack of support was because of a general devaluation of the cultural sector by the United Kingdom government. Many of these participants felt unsupported and suggested that, compared to other sectors, the arts in particular were not a priority (*“I don’t believe that at its core, this government cares about it at all …and I think that the danger is there’s not a consensus that restoring the arts is at the top of anybody’s priority list,” ID6_musician*). As a result, some reported feelings of increasing despair and helplessness:

*“So now I think I feel obsolete, I feel redundant, I feel abandoned, I feel hopeless. I feel that me and my family don’t matter….now that I need help I’m not being given any, and I am angry. I feel disenfranchised” (ID11_independent production associate – community arts and film)*

However, some participants embodied a hope narrative that economic support would be provided by the government in the future (*“The general feeling is that there’s real relief that the government have finally announced some sort of package,” ID9_theatre director*). Consequently, some participants remained optimistic that their financial difficulties would only be short term. This optimism also extended to a perception that economic support for the cultural sector at large would alter public perceptions, resulting in increased funds and “exciting” artistic outputs:

*“I hope that there’s a shift in appreciation of it and hopefully, that will manifest itself in more money and more funding being available and therefore, more work and more people accessing the arts and things like that. I am hopeful for the future, actually and I do think there will be some exciting things that come out of it” (ID18_film and tv producer)*

### Work Dissonance

The socioeconomic changes resulting from COVID-19 were found to cause not only employment and financial instability, but dissonance associated with working in this context. This related to changes to the work environment (e.g., homeworking) and difficulties in adapting work in response to COVID-19 (e.g., online). Pre-existing financial concerns among most participants were also exacerbated by COVID-19, leading to more general feelings of insecurity. Subsequently, some participants expressed a desire to locate employment outside of the CCI in the hope of greater financial stability. Given that the majority of participants derived meaning and enjoyment from their careers in the cultural industries, this was a source of unease.

#### Homeworking

In response to social distancing regulations, many freelancers were required to work from home. Such work was often at odds with many of their former (and ideal) work activities, including interacting with colleagues and peers (*“everything that I do is with people. It’s pretty much face to face. There’s a limit to the effectiveness of what you can do online,” ID12_musician*) and performing to live audiences (*“the real life for me in terms of being able to perform for people in crowds,” ID13_musician*). The realities of homeworking were also often unconducive to cultural work: childcare, for example, was noted as one significant impediment to workflow. Some participants reported working in the evenings to accommodate this, which presented additional psychological stresses:

*“And all of that, I had to do in the evenings, because that was the only work time I had. So, my work shift would be 8PM until midnight or something, most nights. So, that was additionally stressful” (ID14_theatre producer)*

Some participants also described struggling with tasks and a general lack of purpose while working from home. For some, the inability to fulfil work tasks and be creative led to a general feeling of malaise:

*“But I would say my output since the middle of March has been 10% of what it would normally be. And that’s not a good sense, as I’m sure you can recognise, in that it’s not psychologically supportive” (ID6_musician)*

#### Online Difficulties

Interrelated to the challenges of homeworking, some participants reported attempting to modify work – including performances, meetings, and auditions – to suit online platforms. While this was beneficial in the short term, most participants noted several difficulties. For one, many online performances were often unpaid and therefore had little financial incentive. Some freelancers felt this would potentially “demonetize” future cultural work:

“*So all of that was a bonus, but of course it’s also an anxiety for me, because all this music online is free, which demonetizes what I do, and raises long-term issues as to whether they’ll ever want to pay for anything ever again, because they can get it all free online*” (*ID6*_*musician*)

Second, some participants reported difficulties with attempting to perform or rehearse online. For example, some noted the “stilled” nature of video messaging which limited the spontaneity and impulsiveness required for cultural work. This reduced the quality of outputs achieved through such methods. Consequently, most participants felt that moving cultural work online was not practical:

*“We tried to sing [online] but it just didn’t work because of the latency so we were like yes, we can’t really do that” (ID5_musician)*

Finally, many participants stated the importance of socialising and networking with colleagues. To compensate for lack of face-to-face contact, many freelancers transitioned to online platforms to converse. One participant described how a colleague had set up an online support group to support one another over the lockdown period (*“One of my friends, basically, on a Thursday lunchtime, she has open house in her personal Zoom room, and a group of us just drop in and hang out,” ID17_independent theatre producer and arts administrator*). However, participants conceded that such methods were limited in comparison to physical interactions and support (*“being a tactile human being, I think giving people hugs and things is very important for me certainly,” ID6_musician*). Participants also noted how a lack of opportunities for face-to-face socialising increased feelings of isolation and loneliness:

*“I have been feeling quite lonely, because of not being able to see people in person and that being something I enjoy. Even things like networking professionally or whatever, I enjoy being around people, I like people, and you’ve kind of lost that chance encounter with people” (ID17_independent theatre producer and arts administrator)*

#### Leaving the Cultural Industries

For some, the solution to the challenges brought about by COVID-19 was to consider new forms of employment. This was particularly true of young freelancers or those without financial support in the form of savings or financial assistance from parents/partner. While some maintained optimism about a return to work and therefore saw this as a temporary measure, others described COVID-19 as “decimating” their industry and future career plans (*“I’m scared for myself and singing. For me, I think the nail is already in the coffin with regards to singing and being paid for it,” ID13_musician*). As such, some participants were actively considering other forms of employment, as one musician explained:

*“I’m questioning, should I still be a musician? How is this going to survive? I think especially as a freelancer, who’s got no real working rights as it is, really, in the music industry, is there any point in continuing it? Because although I enjoy it, if there’s no money and there’s no work happening for at least a year and a half, it’s like how are you meant to sustain that?” (ID1_musician)*

While some welcomed the stability of “9-5 jobs” (*“I certainly have been considering teaching more full-time and to be quite honest I’m okay with that,” ID8_musician*), others reported unease at potentially transitioning into alternative industries. Most participants described working in the cultural sector in favourable terms, reporting that cultural work was essential to their self-identity: many defined themselves through such work (*“I definitely am aware that I lot of my identity is my work and the work I do,” ID2_film and tv producer*). As a result, most participants showed little motivation for alternative, non-creative forms of employment. Most reported that this would result in the loss of many of the things they enjoyed about freelance cultural work, including the ability to express oneself creatively (*“Ultimately, you’re exploring yourself and what it means to be human and what makes us laugh and what makes us cry…it’s honestly just playtime,” ID10_stage actor*), autonomy (*“I can dictate what I work on and who I work with and how long I work on it for, which I really, really love and would not give that up,” ID16_dance teacher*), and enjoyment (*“We do it because we love it,” ID11_independent production associate – community arts and film*). One freelancer felt that that this would also erode any previous hard work that had enabled him to reach this stage in his career:

*“I trained for this career. I’ve worked very hard in this career. I’ve managed to survive in this career for quite a bit of time. Why should I have to give up my career because of this?” (ID15_stage actor)*

Participants, therefore, reported that thoughts of leaving the cultural sector were a source of stress and unease. For one participant, being unable to perform a job he enjoyed in the future would have psychological implications:

*“Just trying to work out whether that means that I have to renegotiate where I place my purpose, whether that comes from a different career, or whether that comes from staying in what I’m doing…so, there is quite a lot of thinking, I would think I’ve still a lot to do around, and at the moment, it’s probably manifesting itself as generalised anxiety about the future” (ID9_theatre director)*

## Discussion

This study investigated the impact of the COVID-19 pandemic on the working lives of freelancers working in the cultural sector in the United Kingdom and revealed a range of socioeconomic challenges. This included three types of adversity, including employment precarity (including disrupted and reduced workloads), financial instability (including financial hardship), and work dissonance (including transitioning to new forms of employment outside of the sector). These socioeconomic adversities were found to have psychological consequences for participants, including anxiety, loneliness, and psychosomatic symptoms. Our findings thus align with existing research confirming the relationship between socioeconomic insecurity (including unemployment, perceived job insecurity, and financial difficulty) and implications for mental health among the wider population (Jahoda, 1981, 1982; Warr, 1987, 2007; Paul and Moser, 2009; Kim and von dem Knesebeck, 2016; Witteveen and Velthorst, 2020; Wright et al., 2020) and performing arts professionals (Spiro et al., 2021).

In response to COVID-19 restrictions, we found that many participants were subject to either the temporary or permanent loss of employment. Although participants reported pre-existing worries surrounding the precariousness of freelance work, an unfortunate aspect of the COVID-19 crises was the rapid onset of job loss and economic instability: the fact that unemployment was not anticipated meant some participants were unprepared and had little time to plan or find alternative employment. In response, almost all participants who had lost their job reported some form of psychological distress. This is potentially concerning for the wellbeing of freelancers in the cultural sector given that previous research has highlighted the long-term effects of employment transitions (including job loss) on mental health and wellbeing (Thomas et al., 2005; Olesen et al., 2013; Barrech et al., 2016; Herber et al., 2019). Moreover, even if job losses had not occurred, many participants reported worries about future employment. Indeed, it has been suggested that worries about impending adversity are potentially more psychologically demanding than the experience itself (Ferrie et al., 1995). Given the uncertainty surrounding projected timeframes of lockdown measures and a reported lack of control over these circumstances, freelancers may therefore be particularly susceptible to periods of prolonged worrying or rumination about socioeconomic adversity. Many also reported cumulative worries – including financial difficulty and loss of paid work – that are closely related to poor mental health outcomes, including anxiety (Wright et al., 2020).

All participants expressed the importance of work to provide financial resources that could be spent on essentials such as household bills, rent, and existing standards of living. Our findings uncovered how financial hardship following job loss or economic inactivity, however, resulted in difficulties in meeting these needs and a general decline in standards of living. For most participants, this was a source of psychosocial strain and insecurity, a finding generally consistent with literature detailing pathways between financial insecurity and mental health (Paul and Moser, 2009; Witteveen and Velthorst, 2020), including performing arts professionals (Spiro et al., 2021). This includes how inabilities to meet basic needs (such as food, rent, household bills) have implications for mental health through feelings of unease, anxiety, lack of control and insecurity (Butterworth et al., 2009). Our findings contribute to understandings of how COVID-19 induced reductions in income can similarly increase self-reported feelings of mental distress among freelancers working in the cultural sector.

Issues beyond immediate disruptions to employment status and income were also evident. Forms of work dissonance served to destabilise previous work functioning and undermine the working ability of participants. For some, a movement to home working presented a number of work-related stressors, including shared workspaces with family members, childcare, and motivation issues. Relatedly, although most participants were comfortable with and had access to digital technology, the majority noted its incompatibility with forms of cultural work. This included difficulties with remote group performances and a lack of digital connectivity. Consequently, participants described struggling with routine tasks and a general “lack of purpose” while working from home, which was a source of frustration given that most freelancers in the cultural sector derive enjoyment and meaning from their work careers (Gerber, 2017). These changes resulted in an inability to engage in optimal and thus fulfilling work over lockdown periods (Blustein and Guarino, 2020), as evidenced by the general feelings of malaise reported in our findings. This is significant given the centrality of fulfilling forms of work to psychological wellbeing, including its ability to foster feelings of self-esteem, purpose, and autonomy (Jahoda, 1981, 1982; Warr, 1987, 2007; McGann et al., 2016; Blustein and Guarino, 2020).

Changes in working practices, including a migration toward remote and virtual working, may therefore require unique forms of support to ensure it is not only operationally practically but financially viable for freelancers. Some participants noted a potential demonetisation of forms of cultural work in response to COVID-19, particularly in the performing arts industry where traditional, face-to-face performances were superseded by unpaid online performances. Unpaid work is already widespread within the cultural sector and while this may be viable for some – such as those with existing financial security – for younger freelancers or those from lower socioeconomic positions it may serve as a barrier to “getting in and getting on” in the sector (Brook et al., 2018, p. 20). To limit the exacerbation of existing inequalities and to ensure greater diversity and representation in the cultural industries post-COVID-19, support should be tailored to those groups most susceptible to socioeconomic harm.

Many participants noted the destabilisation of social support structures that were previously an important resource for obtaining employment opportunities and assisting in situations of precarity (Brook et al., 2018). The loss of opportunities for socialisation meant support networks were weakened, and this limited both prospects for further employment and opportunities for social support. Some participants detailed how colleagues aided those experiencing psychosocial distress in response to COVID-19 through online groups. This is an important insight for the cultural sector as it is founded upon social networking (Potts et al., 2008), suggesting there may be untapped social resources within the cultural industries themselves that can be instrumental in enhancing coping resources and reducing the experiences of socioeconomic adversity among this group. Harnessing the support of colleagues is especially important given that many participants reported being unable to access financial support intended for those in economic distress (Komorowski and Lewis, 2020). However, some participants noted that the online nature of this social support was insufficient in tackling deeper feelings of psychological distress caused by the circumstances.

Finally, some of our participants also embodied a hope narrative from the government’s pledge of further financial aid, which ultimately prioritised institutions over support for individual freelancers, particularly in England [see Arts Council England [ACE], 2020]. Exploration of precarious work in the classical music industry has found that individuals adopt an entrepreneurial attitude to uncertainty, thereby embracing “the kinds of subjectivities that are forged in order to succeed in the cultural and creative industries” (Scharff, 2017, p. 65). Thus, some of the individual-level “positive” attitudes to “surviving” the economic impact of COVID-19 evidenced in this article may be a similar representation of such logic, which ultimately shifts the burden of responsibility onto the individual and requires them to remain “resilient” in the face of socioeconomic adversity (Comunian and England, 2020). Consequently, individuals may perceive their successes or failures to be the result of individual efforts within a meritocratic system, ultimately masking the need for structural change and leaving freelancers in the sector without the institutional support required to navigate precarious working conditions.

### Limitations

These findings should be considered in light of a number of limitations. First, given the evolving nature of COVID-19 and constant revision of accompanying lockdown restrictions, it is likely that the views and experiences of participants will vary depending on when they were interviewed. Interviews were conducted between July and November 2020, shortly after the initial lockdown period in the United Kingdom (March–May 2020), and therefore do not capture experiences outside of this timeframe. Second, our sample was composed of freelancers from England, Scotland, and Wales. The United Kingdom and devolved governments have coordinated different lockdown responses to COVID-19 within their areas of responsibility, and the views and experiences of participants are therefore likely to differ depending on imposed restrictions and regulations within each administration. Relatedly, funding for freelancers in the cultural sector was distributed differently in each devolved nation, and it is possible that greater financial uncertainty occurred among freelancers in England given that Scotland and Wales provided specific support (such as the Creative Scotland bridging bursary) for those not eligible for self-employment grants. As the majority of participants came from England, however, we cannot predict whether our results would have been different if we included a more geographically diverse sample. Third, the study is limited by the fact that the sample is biassed in favour of interviewees who were motivated or willing to participate. This is a consequence of the convenience sampling method used which, although has allowed for the rapid recruitment of participants during the first stage of the pandemic, is vulnerable to selection bias. It is therefore probable that the views and experiences of those unable or unwilling to participate differ from those documented in this study (e.g., unaffected by COVID-19 restrictions, not worried by job loss) and have therefore not been represented in this study. Relatedly, our data covers a range of professions within the cultural industries, but not all professions. The breadth of different experiences may limit the specificity of the findings as those employed in the theatre industry may have been impacted in different ways to those working in the film and television industry, for example. Finally, although we have recruited from industries where freelance workers comprise a large proportion of the workforce, we were unable to do so from sectors where freelancing is less common (e.g., museums and galleries) (DCMS, 2021b). Conclusions drawn from the research must therefore be considered within the limitations of convenience sampling and should not be extrapolated to the whole population of freelancers working within the cultural sector. Further investigation that encompasses both a broader range of experiences and deeper recognition of the nuances of each industry is therefore needed. Future research that considers the long-term consequences of the pandemic is also required.

## Conclusion

Our study found that participants experienced a range of socioeconomic adversities in response to COVID-19 restrictions, which had psychological consequences for participants, including anxiety, loneliness, and psychosomatic symptoms. The implications of this for the cultural sector, policymakers, and health care services during the ongoing pandemic and beyond are numerous.

Firstly, financial aid is required to support those in the sector facing economic hardship, particularly those working in the performing arts, visual arts, and film and television industries who have been unable to work in the current circumstances or who have been ineligible for economic support. This is necessary not only to support those experiencing deleterious mental health impacts in response to socioeconomic insecurity, but to ensure pre-existing disparities within the sector are not exacerbated. Indeed, it is possible that those able to withstand economic hardship in response to COVID-19 are likely to be those with existing financial resources or safety nets (Brook et al., 2018). It is therefore imperative that those most acutely affected are supported to ensure future inclusivity in the sector. Second, given that our findings provide further evidence of the consistent association between socioeconomic insecurity (including unemployment, perceived job insecurity, and financial difficulty) and implications for mental health (Jahoda, 1981, 1982; Paul and Moser, 2009; Kim and von dem Knesebeck, 2016; Wright et al., 2020), the provision of specific and tailored wellbeing support should be extended to those subject to socioeconomic adversity in attempts to support adaptive coping strategies. This is especially important for freelancers working in the cultural industries given their increased exposure to prolonged periods of economic inactivity and vulnerability to financial shocks, as evidenced previously (Brook et al., 2018; Banks, 2020) and in this study. Relatedly, our research highlights the importance of social networks in providing reassurance and social support during periods of economic instability. This suggests that freelancers may benefit from online interventions that leverage the social and psychological support of peers in attempts to buffer negative psychological consequences. Finally, while these measures may help in mitigating the immediate socioeconomic effects of the pandemic, it is worth noting that many of the issues highlighted in this article existed well before the advent of COVID-19 (Brook et al., 2018; Banks, 2020). Hence, although economic and wellbeing support for freelancers is needed now more than ever, it is important that packages of support are provided beyond this period to address long-standing structural problems within the cultural sector, including the impact of socioeconomic precarity on its workforce.

## Data Availability Statement

The datasets presented in this article are not readily available because they contain information that could compromise the privacy of research participants. Requests to access the datasets should be directed to DF, d.fancourt@ucl.ac.uk.

## Ethics Statement

The studies involving human participants were reviewed and approved by the University College London Research Ethics Committee (Project ID 14895/005). The patients/participants provided their written informed consent to participate in this study.

## Author Contributions

AB and DF contributed to the conception and design of the study. TM was responsible for data collection and wrote the first draft of the manuscript. TM and KW performed the formal data analysis. AB, DF, and KW assisted with review and editing. All authors contributed to manuscript revision, read, and approved the submitted version.

## Author Disclaimer

This COVID-19 Social Study was funded by the Nuffield Foundation (WEL/FR-000022583), but the views expressed here are those of the authors.

## Conflict of Interest

The authors declare that the research was conducted in the absence of any commercial or financial relationships that could be construed as a potential conflict of interest.

## Publisher’s Note

All claims expressed in this article are solely those of the authors and do not necessarily represent those of their affiliated organizations, or those of the publisher, the editors and the reviewers. Any product that may be evaluated in this article, or claim that may be made by its manufacturer, is not guaranteed or endorsed by the publisher.

## Footnotes

1 We follow the United Kingdom government’s Department for Digital, Culture, Media and Sport’s definition of the cultural sector as “those industries with a cultural object at the centre of the industry”; examples include performing arts, visual arts, museums, and galleries, and film, television, and radio (DCMS, 2021a).
